# Adherence to clinical guidelines in integration of mental health services into primary health care in Mbarara, southwestern Uganda: a medical records review

**DOI:** 10.1186/s13033-021-00488-6

**Published:** 2021-07-15

**Authors:** Edith K. Wakida, Moses Ocan, Godfrey Z. Rukundo, Samuel Maling, Peter Ssebutinde, Elialilia S. Okello, Zohray M. Talib, Celestino Obua

**Affiliations:** 1 Department of Psychiatry, Mbarara University of Science and Technology, P. O. Box 1410, Mbarara, Uganda; 2 Department of Pharmacology and Therapeutics, Makerere University College of Health Sciences, Kampala, Uganda; 3 Department of Pharmacology , Mbarara University of Science and Technology, Mbarara, Uganda; 4 Mbarara, Uganda; 5 Mwanza Intervention Trials Unit, Mwanza, Tanzania; 6 Department of Medical Education, California University of Science and Medicine, San Bernardino, CA USA; 7 Office of the Vice Chancellor, Mbarara University of Science and Technology, Mbarara, Uganda

**Keywords:** Uganda Clinical Guidelines (UCG), Adherence, PHC providers, Health management information system

## Abstract

**Background:**

The Ugandan Ministry of Health decentralized mental healthcare to the district level; developed the Uganda Clinical Guidelines (UCG); and trained primary health care (PHC) providers in identification, management, and referral of individuals with common mental disorders. This was intended to promote integration of mental health services into PHC in the country. ‘Common mental disorders’ here refers to mental, neurological and substance use conditions as indicated in the UCG. However, the extent of integration of mental health into general healthcare remains unknown. This study aimed to establish the level of adherence of PHC providers to the UCG in the identification and management of mental disorders.

**Methods:**

This was a prospective medical record review of patient information collected in November and December 2018, and March and April 2019 at two health centers (III and IV) in southwestern Uganda. Data (health facility level; sex and age of the patient; and mental disorder diagnosis, management) was collected using a checklist. Continuous data was analyzed using means and standard deviation while categorical data was analyzed using Chi-square. Multivariable logistic regression analysis was performed to establish predictors of PHC provider adherence to the clinical guidelines on integration of mental health services into PHC. The analysis was conducted at a 95% level of significance.

**Results:**

Of the 6093 records of patients at the study health facilities during the study period, 146 (2.4%) had a mental or neurological disorder diagnosis. The commonly diagnosed disorders were epilepsy 91 (1.5%) and bipolar 25 (0.4%). The most prescribed medications were carbamazepine 65 (44.5%), and phenobarbital 26 (17.8%). The medicines inappropriately prescribed at health center III for a mental diagnosis included chlorpromazine for epilepsy 3 (2.1%) and haloperidol for epilepsy 1 (0.7%). Female gender (aOR: 0.52, 95% CI 0.39–0.69) and age 61+ years (aOR: 3.02, 95% CI 1.40–6.49) were predictors of a mental disorder entry into the HMIS register.

**Conclusion:**

There was a noticeable change of practice by PHC providers in integrating mental health services in routine care as reflected by the rise in the number of mental disorders diagnosed and treated and entered into the modified paper based HMIS registers.

## Background

Mental health disorders, such as depression and substance use, are highly disabling globally [1], and account for 13% of the disease burden in Uganda [1–4]. In 2000, the Ugandan Ministry of Health (UMOH) decentralized mental health delivery to district level [5, 6]; developed the Uganda Clinical Guidelines (UCG) as a practical tool for health workers on the management of common disorders including mental health [7, 8]; and trained primary health care (PHC) providers in identification, management and referral of common mental disorders (i.e., depression, bipolar, epilepsy and alcohol dependence) [6]. Provision of mental health services begins at health center (HC) III (sub-county level) and with subsequent referrals to HC IV (county level), district hospitals, regional referral hospitals and finally to the national referral hospital [8, 9]. Each health facility level (except HC III) is expected to have general doctors (medical officers), clinical officers (Diploma level Medical Assistants), nurses and midwives, and psychiatric nurses.

In 2018, we conducted a single district study to identify the PHC providers perceived barriers to mental health integration and possible solutions. Key barriers included inadequate knowledge/training in mental health, the onerously long and dense format of the UCG, and no provision for recording mental health indicators in patient’s charts [10, 11]. Suggested solutions included provision of a user-friendly summarized UCG for mental disorders, in-service training, and support supervision for mental health [10]. We developed, piloted, and evaluated a 3-component intervention to enhance the capacity of PHC providers in mental health integration for feasibility and acceptability. The components included: a summarized and visually focused version of the UCG on the common mental disorders (i.e., depression, bipolar, epilepsy, and alcohol dependence); modification of existing UMOH paper-based health management information system (HMIS) registers for patient charting to include the common mental disorders; and provision of training and support supervision (by psychiatrists) on the utilization of the UCG algorithm (i.e., diagnosis, management, referral) on the selected disorders. The study showed that the summarized UCG was perceived as user-friendly, and patients were registered for mental healthcare. This was attributed to the training and support supervision they received from the psychiatrists [12]. Non-affective psychosis was not part of the training package, as they were not indicated as common mental disorders; besides, patients are less likely to present to PHC for treatment of psychotic symptoms. The ones who report requiring medical treatment are usually referred to higher level health facilities (regional and national referral hospitals) that have staff with specialist training in mental health.

According to the Uganda Clinical Guidelines (UCG) not all mental disorders are intended to be managed the same way at different PHC levels. There are specific instructions for HC III and HC IV levels, it is expected that empirical treatment is provided at HC III level while specific treatment at HC IV. For example, a patient with signs and symptoms of depression who presents at a HC III level should receive psychosocial support and then be referred to HC IV for further management. Similarly, treatment of bipolar disorder is limited to anti-psychotics (chlorpromazine or haloperidol) at HC III level, while mood stabilizers (carbamazepine) are to be given at HC IV level [7, 8]. In the Ugandan health care system, HC III level facilities do not have general doctor, and so it is not expected that diagnosis and initiation of treatment for bipolar disorder is done at that level.

This review aimed to examine the nature of mental health care and the adherence of PHC to the UCG within primary health care in Mbarara district, southwestern Uganda. Of interest was the prevalence of mental disorders diagnosis during the intervention (presence of psychiatrists providing training and support supervision—November and December 2018) and two months after the psychiatrists training and support supervision (March and April 2019) when the PHC providers have gone back to normal practice; and the alignment of medication with the guideline recommendations for each health facility level.

## Methods

### Study design and setting

This was a prospective medical record review study of patient information collected in November and December 2018, and March and April 2019 at two health centers (III and IV) in Mbarara.

### Study population

The study focused on the primary healthcare (PHC) providers in relation to mental health services offered at PHC level. All the PHC providers who directly assess patients at the selected health facilities were included (i.e., medical officers, clinical officers, nurses, and midwives in routine care).

### Data collection

#### Source of data

The primary source of data was the modified Ministry of Health paper-based Health Management Information System (HMIS) registers for patient charting. The modification involved inclusion of common mental disorders (depression, bipolar, epilepsy and substance use disorders) into the existing paper-based registers. The details of the modifications and other intervention components can be accessed from our previous publication [12]. We reviewed medical records covering a period of six months (i.e., November–December 2018) during intervention when the psychiatrists were providing training and support supervision, and March–April 2019 post intervention. The months of January and February 2019 were excluded from the review to cater for the adjustment of the PHC providers without training and or support supervision from the psychiatrists and to give time for the PHCs to internalize and adapt to using the intervention materials (modified registers and summarized guidelines). Only records with a new mental disorder diagnosis were included for review. Patient records related to follow up visits or for medication refill were also excluded.

#### Data collection procedure

All patient entries in the paper based HMIS registers that were recorded during the study period were de-identified and transformed to an electronic version (Microsoft Access). Data was then extracted using a checklist which had the following items: health facility level; sex and age of the patient; and mental diagnosis, treatment, and management. The checklist was developed based on the intended study outcomes. Two research assistants (one social scientist and one psychiatric nurse) were trained on the data collection tool by the evaluation team which included the psychiatrists who conducted the training in the prior study. On each data collection day, the research assistants reviewed the HMIS forms and extracted relevant data using the checklist. At the end of each data collection day, the study principal investigator (EW) reviewed extracted data from the two reviewers (PK and CA) for correctness and completeness. Instances of incomplete and/or conflicting data were resolved through discussion and any further disagreement was settled through reanalyzing the registers. Entries into the HMIS registers that did not have the patient’s meta-data were excluded.

### Data management and analysis

The Microsoft Access data entry form had checks for quality control. The data was then exported to IBM SPSS *ver 20* for analysis. Continuous data was analyzed using means and standard deviation while categorical data was analyzed using Chi-square. Multivariable logistic regression analysis was performed to establish predictors of PHC provider adherence to the clinical guidelines on integration of mental health services into PHC. The analysis was conducted at a 95% level of significance.

## Results

### Medical records and participant characteristics

A total of 6093 records 2027 (33.3%) from HC III level, and 4066 (66.7%) from HC IV were entered in the Microsoft Access data entry form. Of these 5948 (97.6%) did not have any mental health related entry. The records entered were nearly equal in number—2977 (48.9%) during the intervention period (November to December 2018), and 3116 (51.1%) during the post-intervention period (March and April 2019).

Majority, 4259 (69.9%) of the entries were of female patients. The mean age of the patients was 30.3 ± 21. Over half, 3517 (57.7%) of the patients were aged 19–60 years (Table 1).

**Table 1:** Participant characteristics

Characteristics	Description	Frequency n (%)
Health Facility	HC III level	4066 (66.7)
	HC IV level	2027 (33.3)
Intervention period	During	2977 (48.9)
	Post	3116 (51.1)
Age category (years)	1–18	1956 (32.1)
	19–60	3517 (57.7)
	61+	620 (10.2)
Sex	Female	4259 (69.9)
	Male	1834 (30.1)
Mental illness	Yes	145 (2.4)
	No	5948 (97.6)
Mental diagnosis	Depression	16 (0.3)
	Epilepsy	91 (1.5)
	Bipolar	25 (0.4)
	Alcohol use	14 (0.2)

### Prevalence of mental disorder diagnoses in the health facilities

Of the 6093 records reviewed at the study health facilities during the study period, 146 (2.4%) had one of the four common mental health disorders. Out of the 146 records, 76 were recorded during the intervention when the psychiatrists were offering training and support supervision, and 70 recorded after the intervention (in the fifth and sixth month of the study period).

Of those whose mental disorders were identified, 66/1834 (3.4%) were males compared to females 80/4259 (1.9%), 87 (2.1%) were from HC IV level, and 91 (2.6%) were 19–60 years (Table 2). Of the 146 records, 91 (62.3%) were epilepsy, 25 (17.1%) bipolar, 16 (11%) depression, and 14 (10.6%) alcohol dependence.

**Table 2:** Participant characteristics and mental disorder diagnoses

Characteristics	Mental disorder diagnosis (n = 146)					Total
	Description	Epilepsy	Bipolar	Depression	Alcohol	
Intervention period	During	48 (32.9%)	9 (6.1%)	8 (5.5%)	11 (7.5%)	76 (52.1%)
	Post	43 (29.4%)	16 (10.9%)	8 (5.5%)	3 (2.1%)	70 (47.9%)
Health facility level	HC III	30 (20.5%)	9 (6.1%)	13 (9%)	7 (4.8%)	59 (40.4%)
	HC IV	61 (41.8%)	16 (10.9%)	3 (2.1%)	7 (4.8%)	87 (59.6%)
Sex	Female	50 (34.2%)	14 (9.6%)	12 (8.2%)	4 (2.7%)	80 (54.8%)
	Male	41 (28.1%)	11 (7.5%)	4 (2.7%)	10 (6.9%)	66 (45.2%)
Age category	1–18	35 (24%)	4 (2.7%)	4 (2.7%)	3 (2.1%)	46 (31.5%)
	19–60	51 (34.9%)	21 (14.4%)	11 (7.5%)	9 (6.1%)	92 (63%)
	61+	5 (3.4%)	–	1 (0.7%)	2 (1.4%)	8 (5.5%)

### Medication appropriateness for mental disorder diagnosis and facility level

The most prescribed medications were carbamazepine 65 (44.5%), and phenobarbital 26 (17.8%). The medicines which were prescribed but not aligned with the UCG were chlorpromazine for epilepsy 3 (2.1%) and haloperidol for epilepsy 1 (0.7%) at HCIII. One of the patients newly diagnosed with depression at HCIII was prescribed only folic acid (Table 3).

**Table 3:** Medications prescribed for mental disorder diagnosis

	Epilepsy	Bipolar	Depression	Alcohol	Total
Amitriptyline	–	2	13	–	15
Chlorpromazine	3	8	1	–	12
Carbamazepine	53	12	–	–	65
Haloperidol	1	3	–	–	4
Phenobarbital	26	–	–	–	26
Phenytoin	8	–	–	–	8
Counselling	–	–	1	14	15
Non psychiatric medicine^a^	–	–	1	–	1

^a^The patient was given only folic acid

Of the 15 amitriptyline prescriptions, 12 were given at HC III facility for patients newly diagnosed with depression.

### Factors associated with mental disorder diagnosis in health centers III and IV

In our study, sex was significantly associated with being diagnosed with a mental illness (p < 0.000). The other factors assessed including health facility type, data collection period, and age category were not associated with being diagnosed with mental illness (p > 0.05).

In the bivariable logistical regression analysis, the factors that were significantly associated with mental illness diagnosis in the health facilities in southwestern Uganda included: Female (p < 0.000, 95% CI 0.42–0.72); Age category 1–18 year (p = 0.02, 95% CI 1.16–4.15); and age category 61+ (p = 0.006, 95% CI 1.28–4.38). Those that were not significant included: Health facility (p = 0.057, 95% CI 0.99–1.70); and Follow-up period (p = 0.19, 95% CI 0.63–1.09).

In the multivariable logistic regression analysis, the predictors of a healthcare worker diagnosing a mental illness included, being female (aOR: 0.52, 95% CI 0.39–0.69) and being of age 61+ years (aOR: 3.02, 95% CI 1.40–6.49) (Table 4).

**Table 4:** Predictors of mental disorders diagnosis

Characteristics	Description	cOR (95% CI)	aOR (95% CI)	p-value
Health facility	HC IV	1.0	1.0	
	HC III	1.29 (0.99–1.70)	1.25 (0.95–1.65)	0.107
Intervention period	During	1.0	1.0	
	Post	0.83 (0.63–1.09)	0.85 (0.64–1.12)	0.248
Age category (years)	19–60	1.0	1.0	
	1–18	2.19 (1.16–4.15)	2.63 (0.96–7.24)	0.060
	61+	2.36 (1.28–4.38)	3.02 (1.40–6.49)	0.005
Sex	Male	1.0	1.0	
	Female	0.55 (0.42–0.78)	0.52 (0.39–0.69)	< 0.000

## Discussion

Our study examined the diagnosis and management of common mental health disorders and PHC compliance with guidelines at a primary health care facility in Western Uganda. Of interest was the prevalence identified during the study period, the predictors of being diagnosed with a mental disorders’ diagnosis, and the appropriateness of the management for these mental disorders (medication given and at what health facility level).

Prior to our intervention, there was hardly any record of mental disorder diagnosis at the selected health centers III and IV in Mbarara district. However, during the intervention, 76 patients with a mental disorder diagnosis were recorded, and 70 after the intervention. This implies that the PHC providers retained the knowledge and practice of assessing for mental disorders, a practice that was not common prior to the intervention. The intervention brought about a change in the behavior of the PHC providers reflecting progress in the integration of mental health services into routine care. Although there was noticeable change in practice of integrating mental health services into routine care by the PHC providers, the number of patients recorded was small in relation to the total number of patients seen during the study period at the health facilities. It is possible that there could be a low prevalence of mental disorders in southwestern Uganda. However, a previous study by Ovuga et al. 2005 done in other rural regions of Uganda (Adjumani—west Nile and Busoga—eastern) reported a prevalence of 17.4% for depression. It is therefore likely that the 2.4% mental disorders diagnosed in our study is not a true reflection of mental disorders in southwestern Uganda. The observed low mental disorder entries into the HMIS register could be due to other mental health disorders not being captured; a bias in the patient population if those with mental health condition have lower utilization rates of care (due to lack of motivation associated with their mental health issues); or insufficient adoption of the intervention amongst the PHC providers.

Despite the improvement of PHC providers in assessing for mental disorders in routine care, there was a misalignment of the practice with the UCG. According to the UCG, mental disorder diagnosis and treatment initiation is expected to be done starting from HC IV level; however, from the records review, there were new mental disorder diagnoses made, and treatment initiation at HC III level where there is neither a medical doctor nor a mental health specialist. The correctness of the diagnosis made, and treatment initiated cannot be confirmed. It is expected that empirical treatment is provided at HC III level while specific treatment based on confirmed diagnosis is at HC IV level. Additionally, it was noted that patients at HC III were given specific treatment as per guidelines while some at HC IV were given treatments that did not comply with the guidelines. The observed misalignment with the guidelines is a reflection of the challenges in the referral system [11]. This was highlighted in our previous study in which PHC providers at HC III level noted that it was pointless for them to refer patients to HC IV where they will be treated with the same medications [12]. Additionally, the empirical treatment of bipolar disorder at HC IV and specific treatment of depression and bipolar disorder at HC III could be attributed to the restrictiveness of the guidelines or inadequate knowledge of the medicines by the PHC providers. To improve access to mental health services, there may be a need to relax the restrictive guidelines to allow PHC providers at HC III level to diagnose and treat mental disorders. This however calls for more training and support supervision by mental health specialists.

While we observed misalignment of the practice of PHC providers in the management of mental disorders, this behavior is unlikely to significantly affect the quality and outcome of care received by the patients. One area of non-compliance was the use of carbamazepine (a mood stabilizer) in the management of bipolar disorder, and amitriptyline for depression which were both prescribed at HC III level. Interestingly, these are correct medicines for the conditions, however, they were prescribed at an inappropriate level as per the UCG. The rationale for the guidelines is likely that providers at HC III may not be able to detect and/or manage the unwanted effects of the prescribed medicines. This is potentially a challenge especially because the adverse effects of the medications used may mirror the symptoms of the mental disorders being managed. With the inadequate referral system, the patients are likely to continue receiving medicines for symptoms that are not due to the mental disorder further worsening the condition. This delay in accessing appropriate care is a common problem especially in low- and middle-income countries where the health care systems are inefficient [13].

Our study found that individuals of age 61 years and above were twice more likely to be diagnosed with a mental disorder than those in the age group 18–60 years. This finding is like those of previous studies which reported an association between mental disorder diagnosis and advancement in age [14]. Older people experience dementia as well as other conditions of aging (i.e., loss, frailty, chronic pain) and are therefore more likely to be diagnosed as having a mental disorder which may explain the findings of our study. The likely misclassification of older patients with a mental disorder diagnosis could be attributed to the low level of training among the PHC providers. This means that with the current drive for integration of mental health services into routine care at PHC level, there is need for additional training and support supervision on related conditions such as dementia in the elderly.

In relation to gender, being female reduced the likelihood of being diagnosed with a mental disorder as the rate of mental disorder diagnosis was higher amongst males compared to females. This is contrary to epidemiological studies that have consistently reported higher rates of depression and anxiety disorders in women [15]. The reason may be due to the PHC provider bias in assessing symptoms. Symptoms of depression in women may be passed off as normal stress-related behaviors given the high burden of care and responsibility women carry in the local communities. On the contrary, a man presenting with depressive symptoms may be seen as abnormal and may trigger an assessment for depression. The prevalence of specific mental disorders does differ according to gender. For example, there is higher prevalence of depression and anxiety among women than men; on the other hand, men consistently show higher rates of substance and antisocial disorders [15, 16]. Females are more likely to express their underlying emotional distress compared to men and seek health care. Furthermore, with more females seeking health care compared to males, it is likely that there is significant underdiagnosis of mental health disorders of men in communities [17]. This finding points to the need for the establishment of health promotion interventions targeting a wider segment of the population, to increase awareness, reduce stigma and to empower men and women to seek mental health services.

## Conclusion

Despite the noticeable change in practice of PHC providers in integrating mental health services in routine care as reflected by the rise in the number of mental disorders diagnosed and treated, and entered into the modified paper based HMIS registers, there was some notable nonadherence to the UCG at HC III level. There is a need for the UMOH review the guidelines, their feasibility and acceptability and to consider relaxing the restrictive guidelines to allow PHC providers at HC III level to diagnose and treat mental disorders. Furthermore, there is a need to incorporate mental disorders into the standard paper based HMIS registers used at the health facilities to help act as cues for the PHC providers in assessing for mental disorders in routine care. We recommend additional studies to determine the need for review of medications protocols in the clinical guidelines.

## Data Availability

All data will be available upon request.

## Abbreviations

HC: Health center
HMIS: Health information management system
PHC: Primary health care
UCG: Uganda Clinical Guidelines
UMOH: Uganda Ministry of Health
